# Supplementary material for: Dissecting Stemness in Aggressive Intracranial Meningiomas: Prognostic Role of SOX2 Expression
Source: Int J Mol Sci. 2022 Oct 2;23(19):11690. doi: 10.3390/ijms231911690 (PMC9570252; doi:10.3390/ijms231911690)
Supplement: Supplementary file 1 [file ijms-23-11690-s001.zip › Supplementary Figure S1.pdf]

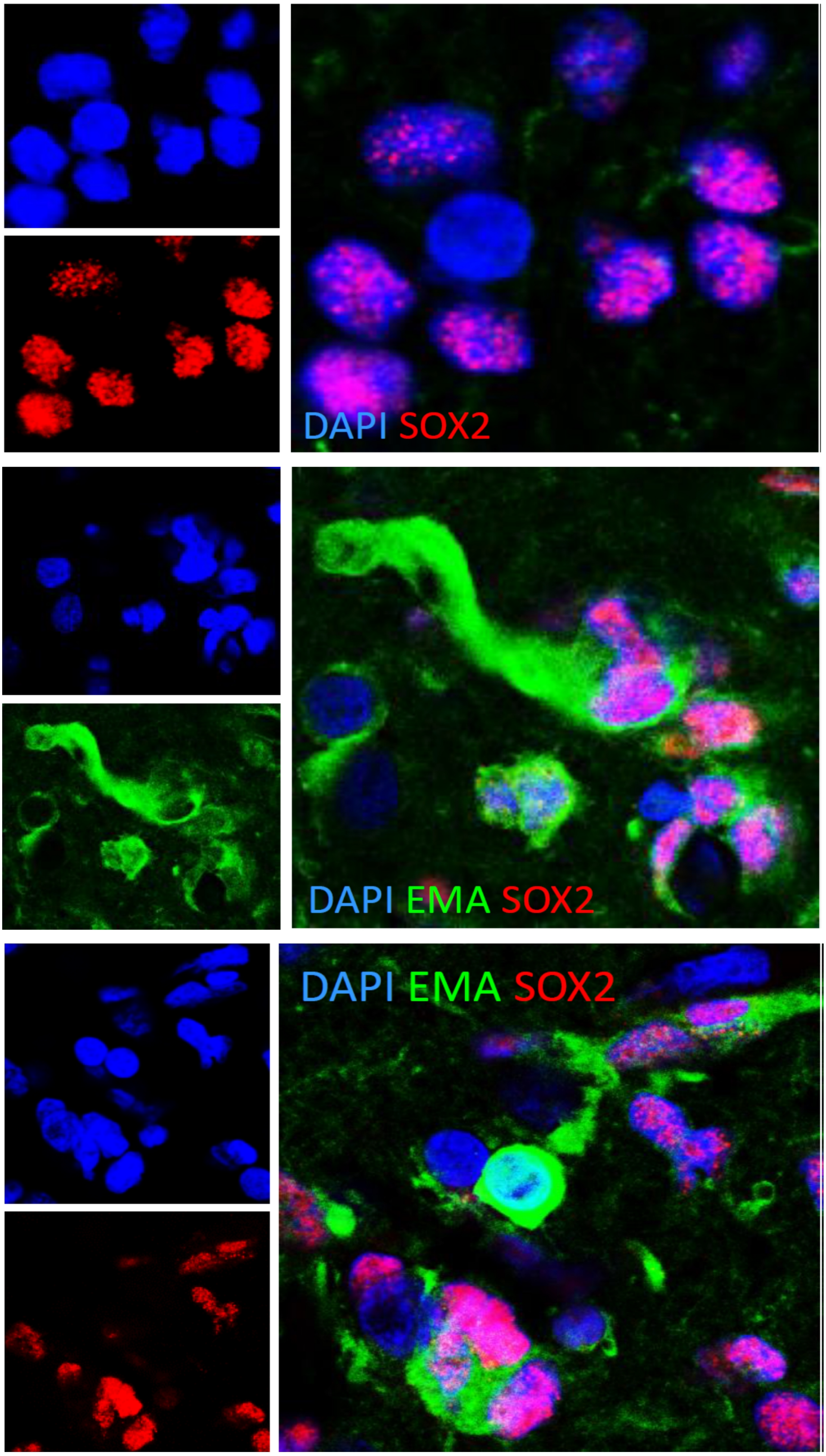

Supplementary Figure S1. Immunofluorescence for SOX2. *Upper row*. Microphotograph showing an atypical meningioma (grade 2) after nuclear staining with DAPI (blue). SOX2 (red) localizes in the cell nuclei. Original magnification 40x. *Middle row*. Microphotograph showing an anaplastic meningioma (grade 3) after staining with anti-SOX2 antibody (red) and with the antibody against the epithelial membrane antigen (EMA), a cell marker for meningioma (green). Most of the tumor cells express SOX2 in their nuclei (DAPI, blue). Original magnification 40x. *Lower row.* Microphotograph showing an anaplastic meningioma (grade III). Some tumor cells have lost their EMA expression as de-differentiation process. Original magnification 40x.
