# Supplementary material for: Dissecting Stemness in Aggressive Intracranial Meningiomas: Prognostic Role of SOX2 Expression
Source: Int J Mol Sci. 2022 Oct 2;23(19):11690. doi: 10.3390/ijms231911690 (PMC9570252; doi:10.3390/ijms231911690)
Supplement: Supplementary file 1 [file ijms-23-11690-s001.zip › Supplementary Figure S2.pdf]

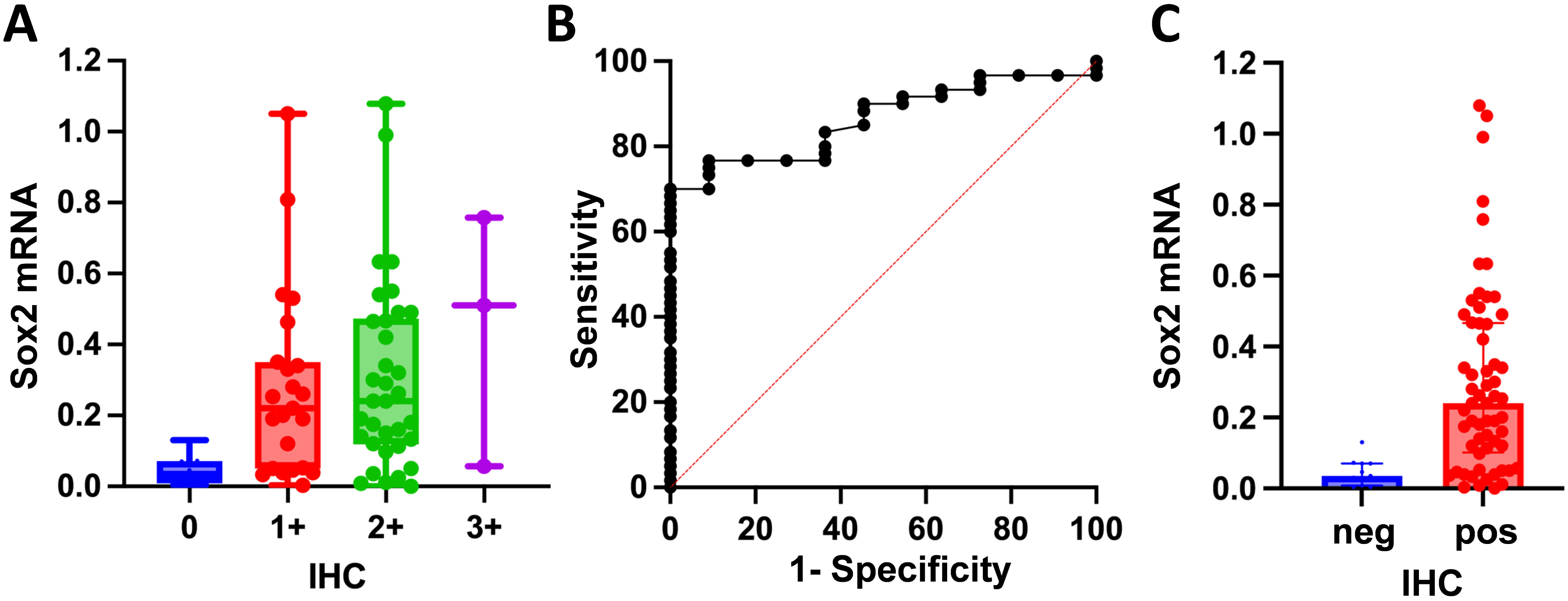

Supplementary Figure S2. SOX2 assessment using IHC and RT-qPCR. *A,* Box plot showing the correlation between SOX2 IHC expression and SOX2 mRNA levels measured with RT-PCR. *B*, ROC curve assessing the accuracy of RT-qPCR mRNA quantification in predicting ICH positivity. *C,* Box plot showing the different SOX2 mRNA levels between positive and negative cases at IHC (***, p<0.001).
